# 

**Published:** 1896-09

**Authors:** 


### PERSONAL.

Dr. H. A. Meisner, of Baltimore, Md., recently officiated as associate judge at the Herring Run Track.

H. B. Adair, of Kansas City, Mo., has successfully passed the civil-service examination, and is on the eligible list for appointment to an inspectorship in the Bureau of Animal Industry.

F. W. O'Brien, V.S., of Hannibal, Mo., entered the civil-service examination for inspectorship in the Bureau of Animal Industry, and has been placed on the eligible list for appointment.

Dr. T. J. Turner has lost none of his interest in Association work, and as President of the Missouri State Veterinary Medical Association has been doing yeoman service in the way of strengthening that organization.

Governor Hastings, of Pennsylvania, has appointed to free scholarships at the Veterinary Department of the University of Pennsylvania Edward Black, of Philadelphia, and John P. Miller, of Lebanon.

Dr. R. S. Huidekoper still remains in the White Mountains, where he is endeavoring to regain his impaired health.

Dr. W. H. Park, assistant bacteriologist of the Health Department of New York City, has been a victim of typhoid fever, contracted while experimenting with toxin intended to be injected in the horse, in the hope of producing an antitoxin for this disease.

Veterinarian Richards, of Emporia, Kan., is an admirer and breeder of English fox-terriers.

Veterinarian J. Greer, of Ormstown, Quebec, has located at Colorado Springs, Colorado.

Veterinarian S. J. Thompson, of Manitoba, holds a governmental place for his people.

Dr. Austin Peters officiated as veterinarian to the Bay State Agricultural Society, whose annual exhibition was held at Worcester, Mass., September 1st to 4th, inclusive.

Dr. William J. Coates, of the American Veterinary College, while alighting from a Lexington Avenue cable-car at 116th Street, New York City, accidentally fell and broke his right arm.

Dr. M. J. Dair during the summer season has been practising at Far Rockaway, L. I.

A well-known and prominent Eastern veterinarian will be among the "Benedicts" next month. Our congratulations later.

Veterinarian H. R. Macaulay, of Indianapolis, has gone to Montreal to study human medicine.

Veterinarian Mull, of Woodburn Farm, Ky., met with a painful accident recently by being thrown from his sulky.

Dr. M. E. Knowles, late of Riverside, Montana, has returned to his old home at Terre Haute, Ind.

Veterinarian Pearson, of Pennsylvania, was a visitor to the Wernersville Sanitarium for a few days in search of rest and recuperation from a severe attack of digestive trouble incidental to the very hot weather.

Dr. Thomas B. Rayner, of Chestnut Hill, Philadelphia, has been suffering from a severe attack of rheumatism of the lower limbs.

